# Using wearable sensors during the timed up and go and 6-minute walk test in Spinal Bulbar Muscular Atrophy

**DOI:** 10.1371/journal.pdig.0001416

**Published:** 2026-05-13

**Authors:** Kruthika Doreswamy, Henry Roberts, Angela Kokkinis, Galen Joe, Abdullah Alqahtani, Christopher Grunseich, Minal Jain

**Affiliations:** 1 Rehabilitation Medicine Department, Clinical Center, National Institutes of Health,‌‌ Bethesda, Maryland, United States of America; 2 National Institute of Neurological Disorders and Stroke, National Institutes of Health, ‌‌Bethesda, Maryland, United States of America; North Carolina A&T State University: North Carolina Agricultural and Technical State University, UNITED STATES OF AMERICA

## Abstract

Spinal Bulbar Muscular Atrophy (SBMA) is a rare and slowly progressive disease that affects males. The Timed Up and Go (TUG) and 6-minute walk test (6MWT) are used in the clinic to track progression. Recent studies have used wearable sensors to track subtle changes in gait and balance in people with rare disease. The objective of this study was to determine whether inertial sensors could be used in men with SBMA to track changes in gait and balance. Our methods included ten participants with SBMA who completed the TUG and 6MWT while wearing six wearable sensors at the baseline, 6-month, and 12-month follow up visits. Our findings showed that this group of men did not progress in their disease over 12 months as evidenced by the stability of TUG durations, 6MWT distance, and sensor parameters. We observed strong associations between the sensor-derived parameters and both TUG duration and 6MWT distance. The sensors can be used during clinic assessments to measure gait and balance parameters. Further studies examining a larger sample size and at-home monitoring should be considered.

## Author summary

People with spinal bulbar muscular atrophy (SBMA) often develop leg weakness that affects mobility and safety. Wearable sensors can capture detailed gait and turning characteristics that are not reflected in standard functional tests. In this study, men with SBMA completed the Timed Up and Go and six-minute walk test with wearable sensors over 12 months. Sensor-derived parameters aligned with clinic-based measures, and individual trajectories provided insight into walking and balance patterns over time. These findings support incorporating wearable sensors into clinic assessments and encourage future work using home-based monitoring in larger SBMA cohorts

## Introduction

Spinal Bulbar Muscular Atrophy (SBMA), also known as Kennedy’s disease, is a rare, X-linked inherited, and slowly progressive neuromuscular disease that affects males [1]. Symptoms typically begin between 30–50 years of age [2]. SBMA is caused by a CAG repeat expansion in the first exon of the androgen receptor (AR) gene, which encodes the polyglutamine (polyQ) tract [3]. Healthy adults generally have less than 35 CAG repeats, while the average repeat length is 45–46 for individuals with SBMA [4,5]. CAG repeats are inversely correlated with age of onset but are not correlated with the rate of disease progression [5–9].

Evidence has shown that SBMA can be driven by nuclear inclusion of AR protein, and evidence of cytoplasmic toxicity has also been reported [10–13]. In individuals with SBMA, mutant AR proteins contribute to the degeneration of anterior horn cells, dorsal root ganglia, and skeletal muscle [1,14–17].

Men with SBMA typically report tremor and cramping years prior to the onset of weakness [8,18]. Over time, balance and gait are affected, which increases the risk for falls and necessitates the use of an assistive device or wheelchair [5]. Other common features of SBMA include neuropathy and bulbar muscle degeneration (dysphagia, dysarthria, and dysphonia) [19,20]. Bulbar and respiratory weakness can lead to pneumonia and subsequent death [5,21].

Currently, there is no approved treatment available for SBMA. Tools used to assess motor function and strength in individuals with SBMA include timed scales (e.g., Timed Up and Go and Six-Minute Walk Test), the Spinal Bulbar Muscular Atrophy Functional Rating Scale (SBMAFRS), and quantitative muscle strength assessment. However, there are limitations regarding the objectivity of these assessments due to the impact of the examiner’s encouragement, the participant’s motivation, and apprehension of falling. There is a need for reliable, valid, and accurate motor outcome measures to support the development of clinical trials in SBMA.

Over the past twenty years, there has been an increase in the use of wearable inertial sensors. Wearable sensors use accelerometers, gyroscopes and/or barometers to work in concert with the body’s movements [22,23]. Instrumented motor assessments using wearable sensors have captured gait and balance metrics in people with Cervical Dystonia (CD), Duchenne Muscular Dystrophy (DMD), and Myotonic Dystrophy type 1 (DM1), and other neurological disorders [21,24–26]. Using these sensors in individuals with SBMA could provide quantifiable parameters of gait and balance and assist in monitoring the overall progression of motor function.

The objective of this study was to describe gait and balance data collected by the sensors and measure motor performance progression in individuals with SBMA during the timed up and go (TUG) and 6-minute walk test (6MWT) assessments.

## Methods

### Ethics statement

The NIH IRB approved this registered clinical trial NCT04944940 sponsored by the National Institute of Neurological Disorders and Stroke (NINDS). All individuals signed informed consent prior to participation.

### Subjects

This is a longitudinal observational study with primary aims to measure and develop clinical and molecular outcome biomarkers and parameters that correlate with SBMA progression, severity, and ability to predict clinical decline. Recruitment is ongoing, and inclusion criteria include males with a genetically confirmed diagnosis of SBMA, ages 18 years or older, and the ability to travel to our clinic. Participants who are non-ambulatory, have contraindications to MRI, and have used androgen-reducing agents within the past two years are excluded. Because SBMA is caused by a CAG repeat expansion in the androgen receptor (AR) gene, prior androgen-reducing therapy may alter AR signaling and downstream molecular markers; therefore, recent use of androgen-reducing agents was an exclusion criterion to avoid confounding the interpretation of clinical and molecular outcomes. A detailed medical history and physical exam were completed by the principal investigator at each visit.

Our analysis here focuses on data collected by wearable sensors worn by participants with SBMA (n = 10) during the Timed Up and Go and Six Minute Walk-Test at baseline, 6 months, and 12-month follow-up visits, with data extraction completed on July 28, 2023. Demographic information and participant disease characteristics were collected from medical records (Table 1).

**Table 1:** Demographic characteristics of participants with SBMA at baseline.

Variables	SBMA (n = 10)
Age (mean ± SD)	62.2 ± 5.5
Height (m) (mean ± SD)	1.8 ± 0.1
Weight (kg) (mean ± SD)	92.9 ± 19.2
BMI (%)	
Healthy (BMI: 18.5-24.9)	n = 1
Overweight (BMI: 25.0-29.9)	n = 7
Obese (BMI: 30-34.9)	n = 1
Extremely obese (BMI: + 35)	n = 1
Age of Onset (years)	
20-29	n = 1
30-39	n = 1
40-49	n = 6
50-59	n = 2
CAG repeats (mean ± SD)	45.0 ± 3.0
SBMAFRS Score (mean ± SD)	42.0 ± 8.0
Assistive Device Use	5

### Wearable sensors

**Fig 1:**
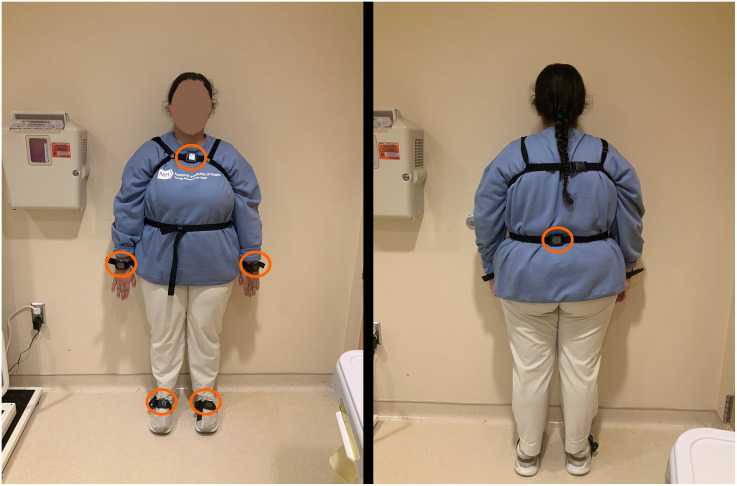
I (MJ) have taken this photo and give permission to PLOS to publish this photo under the CC-BY 4.0 license.

Opal sensors (APDM, Inc.) were used to collect movement data. Each sensor includes a tri-axial accelerometer and a gyroscope to capture linear acceleration and angular velocity. A research physical therapist placed six sensors on participants at each visit: one on each wrist, one on each foot, the sternum, and the lumbar region (Fig 1), following APDM’s standardized protocol. After initialization, participants performed the Timed Up and Go (TUG) and Six-Minute Walk Test (6MWT). Data were recorded at 128 Hz using the MobilityLab2 application and processed with APDM’s proprietary algorithms.

### Timed up and go (TUG)

The TUG is a functional mobility assessment that measures the time it takes a person to stand up from their chair, walk 3 meters, turn around, and sit down [27,28]. The wearable sensors collected the following information: duration(s), turn velocity (degrees/sec), and turn duration (s).

### 6-minute-walk-test (6MWT)

The 6MWT is a reliable primary outcome used to measure a person’s exercise capacity, cardiopulmonary and neuromuscular abilities, and to quantify the progression of neuromuscular diseases [29–32]. It has been proposed as an accurate disease marker for individuals with SBMA, with the ability to capture an approximate 10% decline over 1 year [33]. Heart rate and blood pressure were recorded prior to testing, immediately after testing, and after a 10 min recovery. During this test, the participant walked for 6 minutes along a flat, straight hallway, modifying the American Thoracic Society’s recommended length of 30 meters to 39 meters [30]. The modification was necessary to minimize the number of turns the participant had to complete due to impaired balance and an increased risk for falls. However, this corridor modification may affect direct comparison with published reference equations and normative values that are based on the standard 30-meter corridor. As a result, six-minute walk distances reported in this study should be interpreted with caution when compared to reference equations derived from a 30-meter course. Encouragement was provided every minute. The distance (m) (6MWD) walked was recorded using a measuring wheel. The wearable sensors captured multiple parameters during the 6MWT, including cadence (step/min), stance (%), stride-length (m), and gait cycle duration (s). Stride velocity was calculated using the following equation:


$$stride\;velocity\;\left(\frac{m}{s}\right)=\;\frac{stride-length\;(m)}{gait\;cycle\;duration\;(s)}$$ Equation 1

The 95^th^ percentile of the stride velocities was calculated to provide the stride velocity 95^th^ centile (SV95C).

### SBMA Functional Rating Scale (SBMAFRS)

The SBMAFRS is a recently validated scale for individuals with SBMA and took inspiration from the Revised Amyotrophic Lateral Sclerosis Functional Rating Scale (ALSFRS-R) [34]. This questionnaire includes questions surveying fourteen activities of daily living (ADL); each item is scored from 0 to 4, with 0 indicating the ADL is impossible and 4 indicating no problems with the task, with a total score of 56. A lower score indicates greater challenges in completing functional tasks. Loss of ambulation was determined by the following scores: 4 (No problem with walking/running), 3 (No problem with walking but difficult to run), 2 (A cane or assistive device is sometimes needed), 1 (A cane or assistive device is always needed), and 0 (Walking is impossible) [34]. The baseline scores are reported in Table 1.

### Statistical analysis

Fifteen participants with spinal bulbar muscular atrophy (SBMA) enrolled and completed baseline visits. Ten participants completed all three study timepoints (baseline, 6 months, and 12-months) and were included in the final longitudinal analysis. Five participants were excluded from the longitudinal analysis due to: device malfunction (n = 2), withdrawal from the study (n = 1), and inability to complete 6-month or 12-month follow-up visits (n = 2).

Beyond these participant-level exclusions, missing data for specific sensor parameters occurred at individual visits due to technical issues, even among the 10 participants who completed all three timepoints. Technical failures included sensor initialization errors, loss of wireless connectivity during data collection, premature battery depletion, and post-hoc data processing failures where algorithms could not extract specific parameters (e.g., sit-to-stand transitions) from recorded movement data. Each TUG and 6MWT parameter was analyzed using all available observations for that specific measure, resulting in varying sample sizes across parameters and timepoints as noted in Table 2. We report the rate and nature of missing data as they directly inform the feasibility of implementing these sensors in clinical practice.

**Table 2:** Spinal Bulbar Muscular Atrophy (SBMA): mean TUG and 6MWT scores by select activities and time period.

			Baseline			6-Months			12-Months			(μ_baseline_ = μ_6months_ = μ_12months_)
**Test**	**Activity**	**Metric**	**N**	**Mean**	**S.E.**	**N**	**Mean**	**S.E.**	**N**	**Mean**	**S.E.**	**P-value**
TUG	Turning	Angle (degrees)^a^	9	195.7	22.5	10	171.8	9.6	8	181.5	6.4	0.5273
		Duration(s)^b^	9	10.9	2.3	10	12.4	2.6	9	9.7	1.5	0.5798
		Velocity (degrees/s)^a^	9	221.2	25.9	10	218.1	26.8	8	246.7	32.2	0.7825
	Sit-to-Stand	Duration (s) and Lean Angle (degrees)	7	0.9	0.1	9	1.0	0.1	8	1.0	0.1	0.1676
	Stand-to-Sit	Duration (s) and Lean Angle (degrees)	8	0.7	0.1	9	0.8	0.1	7	0.6	0.1	0.0755
6MWT	Gait	Cadence (steps/min)^c^	10	104.5	6.4	9	103.1	6.5	10	102.5	7.5	0.4747
		Stance (%)^c^	10	62.8	1.1	9	59.7	3.6	10	63.5	1.4	0.3647
		Double Support (%)^c^	10	25.7	2.2	9	26.9	2.3	10	27.0	2.9	0.1823
		Stride-Velocity (m/s)^c^	10	1.2	0.1	9	1.2	0.1	10	1.2	0.2	0.4581
		Stride-Length (m)^c^	10	1.3	0.1	9	1.2	0.1	10	1.2	0.1	0.0686
		6MWT Distanced Walked^b^	10	422.9	51.2	9	443.7	52.36	9	427.0	61.36	0.5377
SBMAFRS Total Scores^b^			10	41.9	2.51	10	40.5	3.25	8	38.5	3.37	0.2767

^a^Obtained from sensors during i-TUG; ^b^Obtained from manual measurement; ^c^Obtained from sensors during i-6MWT;

Linear mixed models (LMMs) were used to determine whether the mean patient (TUG or 6MWT) results (μ _baseline_ = μ _6months_ = μ_12months_) were statistically equal. Subject-specific random intercepts were specified to account for within-subject correlation from repeated measurements and to capture between-participant variability in baseline walking performance. Each model comprised a dependent variable (TUG or 6MWT metric) and an independent variable (visit). For each LMM, an F-test was used to test if the effect for the study time-period was equal to zero, which is comparable to the mean responses being equal for each time-period. Resulting p-values < 0.05 were statistically significant.

Scatter plots and LMMs were used to characterize the association between mean TUG duration and selected TUG parameters (turn angle and turn velocity), and to evaluate whether these associations differed across assessment periods (baseline, 6-months, and 12-months). Each model included TUG duration as the dependent variable, visit, and one TUG parameter (turn angle or turn velocity) as fixed effects, and a visit-by-parameter interaction term. Subject-specific random intercepts were incorporated to account for between-participant variability in baseline TUG duration.

Similarly, scatter plots and LMMs were used to characterize the association between mean 6MWD and selected 6MWT parameters (cadence, stance time, double-support time, stride velocity, and stride length), and to assess whether these associations varied across assessment periods (baseline, 6-months, and 12-months). Each model specified 6MWD as the dependent variable, with visit and one 6MWT parameter included as fixed effects, along with a visit-by-parameter interaction term. Subject-specific random intercepts were incorporated to account for between-participant variability in baseline 6MWD. Fixed-effect estimates are reported with standard errors and p-values, with p-values < 0.05 considered statistically significant.

All analyses were conducted using SAS version 9.4 statistical software.

## Results

### Demographic characteristics

All participants were male with a mean age of 62.2 years (SD = 5.5). The mean CAG repeat sequence was 45.0 (SD = 3.0), and the mean SBMAFRS score at baseline was 41.9 (SD = 8.0). Five (50%) participants used an assistive device for ambulation, such as a cane or a walker (Table 1), but no participant changed their assistive device used during the 12-month follow-up.

### Technical feasibility and missing data

Sensor-related technical issues resulted in substantial data loss across timepoints. Of 30 possible participant visits (10 participants × 3 timepoints), complete sensor data were obtained for only 53–87% of observations, depending on the parameter (Table 2).

For TUG measurements, the most affected parameter was sit-to-stand duration, with only 7/9 baseline measurements (78%), 9/10 six-month measurements (90%), and 8/9 twelve-month measurements (89%) successfully captured. Turn angle and turn velocity were captured in 100% of six-month visits, but only 89% of baseline and 89% of twelve-month visits.

For 6MWT measurements, cadence, stance, double support, stride velocity, and stride length were captured more consistently (90–100% across timepoints), though individual missing observations occurred at each visit.

Technical failures were attributable to sensor initialization errors prior to testing (n = 2 participants excluded from entire analysis), intermittent wireless connectivity loss during data collection, and algorithm failures in post-processing, where specific movement transitions (particularly sit-to-stand and stand-to-sit) could not be reliably identified from recorded signals.

### Analysis of TUG gait and balance data

**Fig 2:**
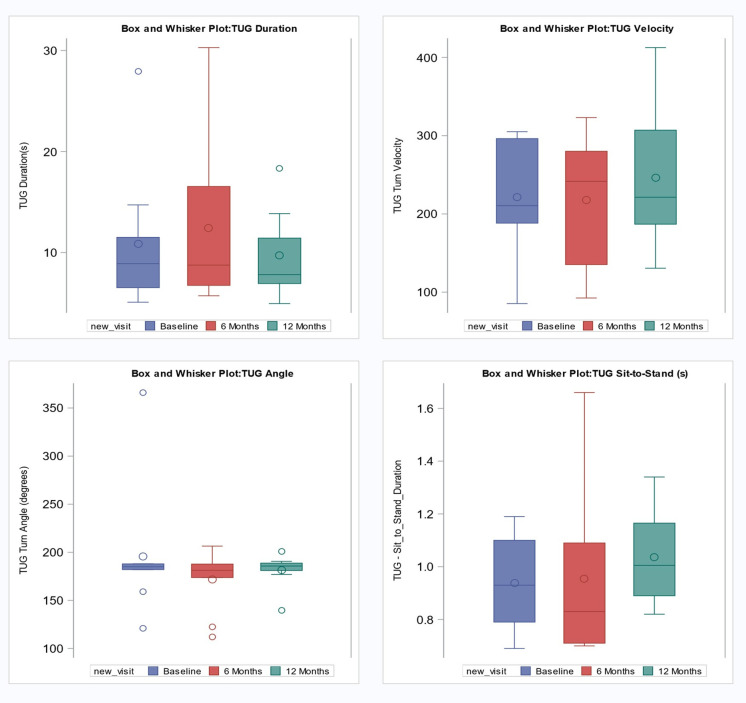
Box and whisker plots showing changes in Timed Up and Go (TUG) test components over a 12-month period. The plots display TUG duration, turn velocity, turn angle, and sit-to-stand duration at baseline, 6-month, and 12-month intervals.

The mean time taken to complete the TUG assessments was approximately 10.9 seconds at baseline, 12.4 seconds at 6-months, and 9.7 seconds at 12-months (Table 2). The interquartile ranges at baseline and 12-months indicate consistent balance and mobility for the middle half of TUG times (Fig 2). Overall, TUG performance remained relatively stable over the three time periods (p-value = 0.58; Table 2). The mean TUG turn velocity was approximately 221.2 degrees per second at baseline, 218.1 degrees per second at 6-months, and 246.7 degrees per second at 12-months (Table 2). The distribution of TUG turn velocity showed overlapping interquartile ranges at baseline, 6-months, and 12-months, with progressively higher maximum values across time points (Fig 2). TUG turn velocity remained relatively stable across the three time periods (p = 0.68; Table 2).

A few individuals exhibited extreme Timed Up and Go (TUG) metrics, identified as statistical outliers with measurements falling beyond the whisker range of the plots. Specifically, Patient #9 recorded the highest TUG duration at both baseline (27.9 seconds) and the 6-month follow-up (30.3 seconds). At the 12-month assessment, Patient #4 showed the highest TUG duration (18.3 seconds). Furthermore, Patient #16 exhibited the highest TUG turn angle at baseline (366.1 degrees/second) and at the 6-month follow-up (206.4 degrees/second). Interestingly, by the 12-month mark, this same patient’s turn angle was among the lowest recorded at 139.8 degrees/second.

**Fig 3:**
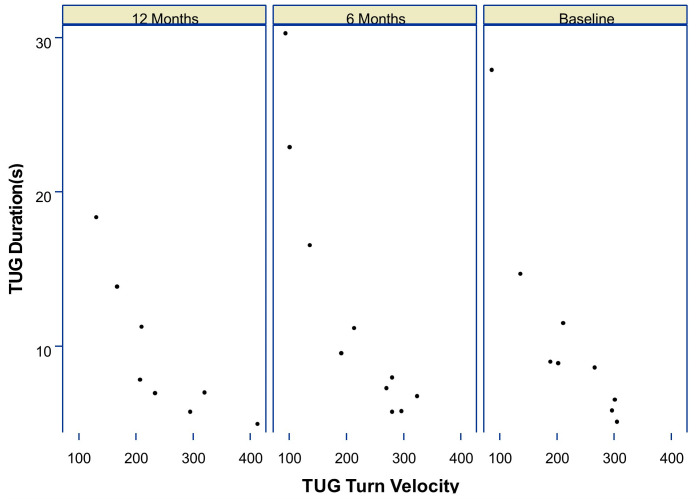
Scatterplot of Timed Up and Go (TUG) Duration versus TUG Turn Velocity. The data is paneled by visit, showing measurements at Baseline, 6 Months, and 12 Months.

TUG duration and turn velocity were inversely related across visits. Examination of the scatter plots in Fig 3 shows that participants who completed the TUG more quickly also tended to exhibit faster turning performance, highlighting a consistent relationship between overall mobility efficiency and turning agility. There was no evidence that the association between TUG duration and turn velocity varied over time. The interaction between turn velocity and visit was not statistically significant at either follow-up assessments (6-months: p-value = 0.34; 12-months: p-value = 0.49), indicating that the relationship between TUG duration and turn velocity remained stable across study visits, Table 3.

**Table 3:** Summary of Linear Mixed-Effects Random Intercept Models Examining Gait Parameters and Their Interactions With Visit on Mean Tug Duration.

Model 1: Predictor	β	SE	p-value	Model 2: Predictor	β	SE	p-value
Intercept	13.4	3.18	0.0004	Intercept	15.54	4.17	0.0013
Turn-Angle_(degrees)_	-0.01	0.01	0.4742	Turn-Velocity_(degrees)_	-0.02	0.02	0.3314
Visit_(12-months vs Baseline)_	-5.2	9.41	0.5905	Visit_(12-months vs Baseline)_	2.78	2.6	0.3185
Visit_(6-months vs Baseline)_	6.94	5.47	0.2261	Visit_(6-months vs Baseline)_	2.66	2.12	0.2536
Visit_(12-months)_ × Turn-Angle_(degrees)_	0..03	0.05	0.5474	Visit_(12- months)_ × Turn-Velocity_(degrees)_	-0.01	0.01	0.4913
Visit_(6-months)_ × Turn-Angle_(degrees)_	-0.04	0.03	0.2380	Visit_(6-months) ×_ Turn-Velocity_(degrees)_	-0.01	0.01	0.3420

Each model adjusted for visit and included a subject-specific random intercept. Visit × gait parameter terms represent time-varying associations with TUG Duration

### Analysis of 6MWT Gait and Balance Data

**Fig 4:**
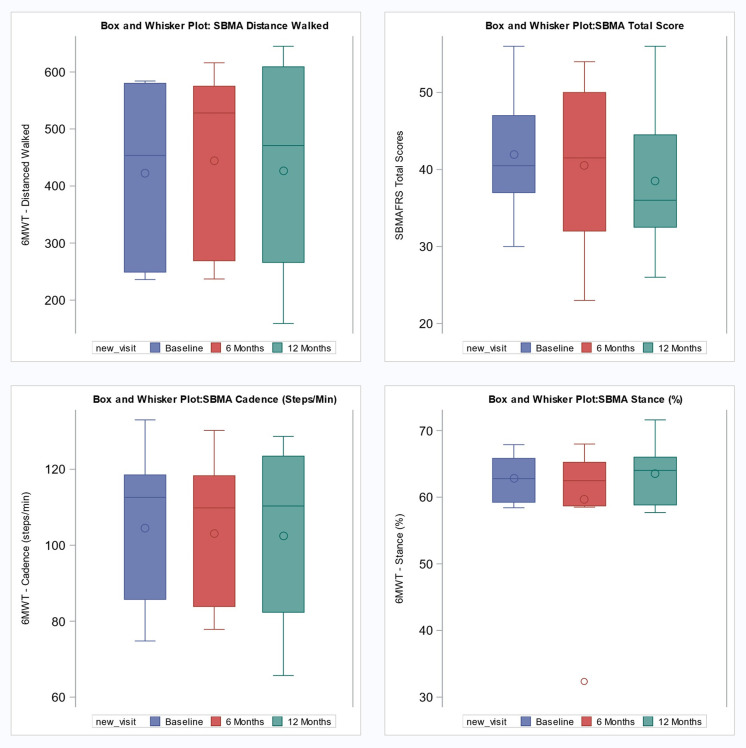
Box and whisker plots showing changes in Total Distance walked, SBMAFRS total Score, SBMA Stance and Cadence at baseline, 6-month, and 12-month intervals.

The mean distance walked during the 6MWT assessments was approximately 422.9 meters at baseline, 443.7 meters at 6-months, and 427.0 meters at 12 months, as shown in Table 2. The interquartile ranges at baseline, 6 months, and 12-months indicate consistent variability in the mean distance walked at each visit (Fig 4). The mean 6MW distances remained relatively stable over the three time periods (p = 0.54; Table 2).

The mean 6MWT cadence was 104.5 steps/min at baseline, decreasing slightly to 103.1 steps/min at 6 months and 102.5 steps/min at 12 months (Table 2). At baseline, the IQR for 6MWT cadence was 85.7–118.5 steps/min, indicating moderate between participant variability. By 12 months, the IQR had widened to 82.3–123.5 steps/min, reflecting a modest increase in cadence variability across the assessment periods (Fig 4). Cadence remained relatively stable across the three assessment periods (p = 0.48; Table 2).

**Fig 5:**
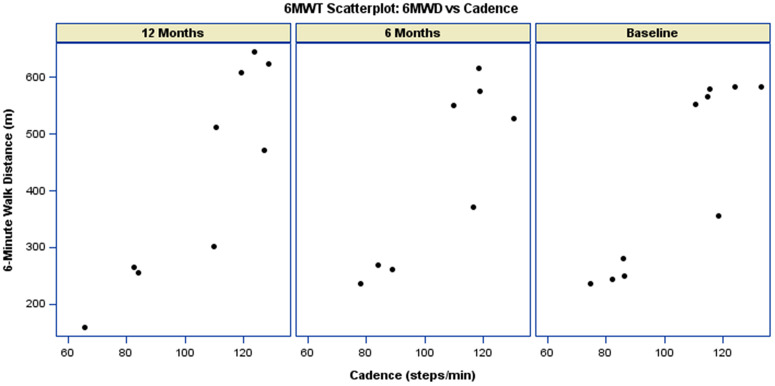
Scatterplot of 6MW distance versus 6MWT Cadence. The data is paneled by visit, showing measurements at Baseline, 6-Months, and 12-Months.

As shown in Fig 5, participants with higher cadence tended to achieve longer walking distances. There was no evidence that the association between cadence and six-minute walk distance (6MWD) varied over time. The interaction between cadence and visit was not statistically significant at either follow-up assessment (6 months: p-value = 0.2625; 12 months: p-value = 0.3854), indicating that the relationship between cadence and 6MWD remained stable across study visits, Table 4.

**Table 4:** Summary of linear mixed-effects random intercept models examining gait parameters and their interactions with visit on mean 6-Minute Walk Distance.

Model 1: Predictor	β	SE	p-value	Model 2: Predictor	β	SE	p-value
Intercept	-391.08	114.65	0.0027	Intercept	564.68	292.06	0.0754
Cadence_(steps/min)_	7.79	1.07	<0.0001	Stance_(%)_	-2.26	4.58	0.6312
Visit_(12-months vs Baseline)_	32.43	46.98	0.5016	Visit_(12-months vs Baseline)_	412.64	245.64	0.1200
Visit_(6-months vs Baseline)_	-58.94	52.26	0.2794	Visit_(6-months vs Baseline)_	-145.94	296.59	0.6314
Visit_(12-months)_ × Cadence_(m/s)_	-0.39	0.43	0.3854	Visit_(12-months)_ × Stance_(%)_	-6.73	3.91	0.1119
Visit_(6-months)_ × Cadence_(m/s)_	0.57	0.49	0.2625	Visit_(6-months)_ ×Stance_(%)_	2.38	4.72	0.6229
**Model 3: Predictor**	**β**	**SE**	**p-value**	**Model 4: Predictor**	**β**	**SE**	**p-value**
Intercept	-67.74	19.11	0.0031	Intercept	-243.41	81.19	0.0099
Stride-Velocity_(m/s)_	405.9	15.04	<0.0001	Stride-Length_(m)_	533.47	63.55	<0.0001
Visit_(12-months vs Baseline)_	12.57	10.66	0.2769	Visit_(12 -months vs Baseline)_	13.15	56.13	0.8191
Visit_(6-months vs Baseline)_	-13.85	8.52	0.1462	Visit_(6-months vs Baseline)_	23.62	32.58	0.4877
Visit_(12-months)_ ×Stride-Velocity_(m/s)_	-16.39	8.08	0.0831	Visit_(12-months)_ ×Stride-Length_(m)_	-2.69	43.12	0.9515
Visit_(6-months)_ ×Stride-Velocity_(m/s)_	9.05	6.6	0.2099	Visit_(6-months)_ ×Stride-Length_(m)_	-16.32	25.68	0.5417

The interquartile range (IQR) of 0.8–1.5 m/s for 6MWT stride velocity at baseline indicates the middle 50% of participants ranged from slow to near-normal velocities. At 12 months, a similar IQR of 0.8–1.6 m/s suggests that overall variability remained stable (Fig 4).

**Fig 6:**
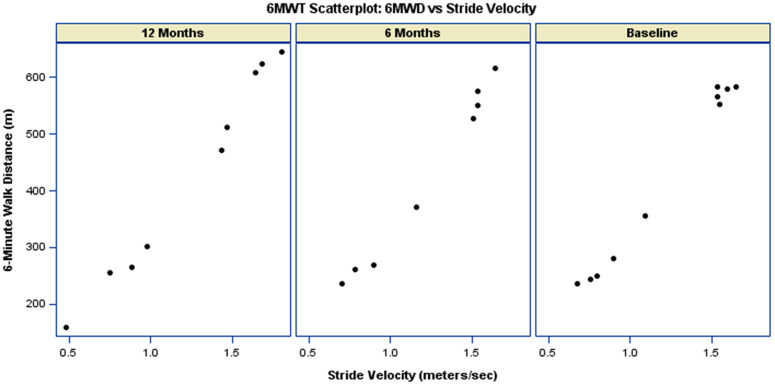
Scatterplot of 6MW distance versus 6MWT Stride Velocity. The data is paneled by visit, showing measurements at Baseline, 6-Months, and 12-Months.

**Fig 7:**
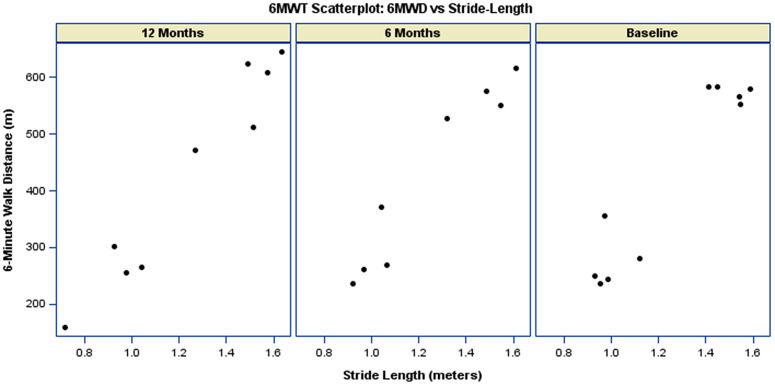
Scatterplot of 6MW distance versus 6MWT Stride Length. The data is paneled by visit.

Participants with higher stride-velocity (m/s) tended to achieve longer walking distances (Fig 6). The average stride velocity remained constant at 1.2 m/s for each of the three time periods (Table 2). As such, the relationship between stride-velocity (m/s) and 6MWD remained stable over visits. As shown in Table 4, the interaction between stride-velocity and visit was not statistically significant at either follow-up assessment (6 months: p-value = 0.21; 12 months: p-value = 0.08). Similar to participants with higher stride-velocity (m/s), participants with higher stride-length (m) tended to achieve longer walking distances (Fig 7). The relationship between stride-length (m) and 6MWD remained stable over visits (Fig 7, Table 4).

The 6MWT sensor parameters (average cadence, average stride velocity, average 6MWD) remained stable over time (baseline, 6-months, and 12-months) (Table 2). The average 6MWT cadence ranged from 102.4 steps/min to 104.5 steps/min and was not statistically significant (p = 0.47). The average stride velocity remained constant at 1.2 m/s for each of the three time periods. The average 6MWD ranged from 422.9m at baseline to 427.0m at 12 months, and the difference between the average 6MWD was not statistically significant (p = 0.46).

### Analysis of 6MWT, SBMAFRS scores, and turn angle data

The mean SBMAFRS score was approximately 41.9 at baseline, 40.5 at 6-months, and 38.5 at 12-months (Table 2). Scores showed no significant change across the three time points (p-value = 0.28; Table 2).

## Discussion

SBMA is a slowly progressive neurodegenerative disease that can impact an individual’s gait, balance, and subsequently affect their activities of daily living. Disease-modifying therapeutics for SBMA are being developed, creating an urgency to identify objective measures to track gait and balance over time, in the clinic and remotely.

At the group level, mean TUG duration and six-minute walk distance showed modest changes over 12 months. The 4% mean decline in 6MWD (95% CI -6.7%, 14.8%) aligns closely with the 4.7% annual decline reported by Dahlqvist et al. in a larger SBMA cohort measured over 18 months. However, the lack of statistical significance in our small sample (n = 10) should not be interpreted as evidence of stability.

Individual trajectories revealed heterogeneity in disease progression (Fig 1 and Fig 2). Three of ten participants (30%) showed a one-point decline on the SBMAFRS walking item between baseline and twelve months, and individual 6MWD changes ranged from improvement to substantial decline. These findings highlight that group-level means and p-values may obscure clinically meaningful changes in small, rare disease cohorts, and that individual-level data and effect sizes provide more informative assessment of disease progression. No participants changed their assistive device use during the 12-month follow-up period, which may have provided stability during structured clinical assessments despite underlying disease progression. A critical finding of this study relates to the technical feasibility of implementing wearable sensors in clinical SBMA assessments. The substantial rate of missing data (13–47% of observations depending on parameter) raises important concerns about the readiness of this technology for routine clinical use. Device initialization failures excluded 13% of enrolled participants entirely, while algorithm failures to extract specific parameters (particularly sit-to-stand/stand-to-sit transitions) affected up to 33% of individual measurements. These technical limitations likely reflect a combination of factors: hardware reliability issues, challenges with wireless data transmission in clinical environments, and algorithm sensitivity to movement variability in individuals with neuromuscular disease. The sit-to-stand transition detection failures may indicate that algorithms developed and validated in healthy populations or other disease populations require specific optimization for SBMA movement patterns, where muscle weakness and tremor may alter the kinematic signatures that these algorithms rely upon.

For clinical trial implementation or routine clinical monitoring, this level of data loss would be problematic. Future work should focus on improving hardware reliability and battery life, developing disease-specific algorithm training sets, implementing real-time data quality monitoring to allow immediate re-testing when failures occur, and establishing clear protocols for handling missing data in longitudinal assessments. Until these technical barriers are addressed, researchers and clinicians should anticipate data loss rates of 15–30% when planning studies using these sensors in SBMA

Previous studies have explored the used of wearable sensors in individuals with CD, DMD, and DM1 [24–26]. Individuals with CD had a significantly higher duration for spatio-temporal parameters collected during the instrumented TUG (i-TUG) in comparison to the control group [24]. There was a moderate correlation between the proportional integration mode (PIM), which is indicative of activity level, and 6MWD (ρ = 0.58, p-value<0.01) [25]. When exploring accelerometers in individuals with DM1, ankle-worn devices provided more reliable results compared to sensors worn at other locations [26].

Individuals with SBMA report difficulties with activities of daily living, which are captured by the SBMAFRS. When examining the correlation between activities of daily living and motor assessments, our study found strong correlations between the SBMAFRS score and the 6MWD, and strong inverse correlations with stance and double support (see Table 5). To bridge the gap between motor capacity and motor performance, we suggest using sensors for home monitoring among individuals with SBMA.

**Table 5:** Correlation between SBMAFRS and sensor parameters.

Parameter	Baseline		6 months		12 months	
	**r** **_s_**	**p**	**r** **_s_**	**p**	**r** **_s_**	**p**
Turn Velocity (degrees/s)^a^	0.70000	0.0358	0.5273	0.1173	0.4685	0.2890
6MWD ^b^	0.7212	0.0186	0.6833	0.0424	0.7857	0.0362
Cadence (steps/min)^c^	0.5394	0.1076	0.4167	0.2646	0.6467	0.0831
Stance (%)^c^	-0.7939	0.0061	0.0000	1.0000	-0.7665	0.0265
Double Support (%)^c^	-0.7939	0.0061	-0.4667	0.2054	-0.7665	0.0265
Stride-Velocity (m/s)^c^	0.7091	0.0197	0.3833	0.3085	0.6587	0.0757
Stride-Length (m)^c^	0.6121	0.0600	0.4167	0.2646	0.6707	0.0687

r_s_ – Spearman’s Correlation Coefficient; ^a^Obtained from the wearable sensors during i-TUG; ^b^Obtained from manual measurement; ^c^Obtained from the wearable sensors during i-6MWT

The use of sensors for home monitoring could provide a more accurate representation of motor function and progression in individuals with SBMA, but no studies have been completed with this population. A study examining home monitoring in individuals with Huntington’s Disease (HD) over a 7-day period found greater variability in gait measures in comparison to measures collected during clinical assessments [35]. In boys with DMD, step activity, measured ‌‌remotely over a 7-day period, was found to be related to and predictive of functional decline [36,37]. Currently, we are working on creating and validating an algorithm for home monitoring in individuals with SBMA. Due to the sample size of ultra-rare and rare diseases, using home monitoring should be explored as a method to monitor the progression of disease.

The European Medicines Agency (EMA) has recently approved the use of stride velocity 95^th^ centile (SV95C) as the first wearable-derived digital clinical outcome measurement in boys with DMD [37]. Data recorded over 180 hours from the clinic visits and home monitoring were used to calculate the SV95C, an EMA-approved secondary endpoint [37]. We recognize that our calculation of SV95C differs from the prolonged home-monitoring paradigm used in regulatory qualification, and therefore, results should not be directly compared with home-based SV95C values. Our plan is to extend these analyses to home monitoring datasets in the continuation of the study.

Examining the changes over the 12-month period, we found that the mean SV95C (m/s) was 1.2 m/s at every visit (p-value = 0.54) (Table 2). When examining participants individually, SV95C remained stable over the 12-month period. Sensor-derived parameters and home monitoring could provide a more accurate and sensitive view of functional changes in the SBMA population.

The strengths of this study include its focus on the natural history of SBMA, a rare, neuromuscular disorder, the use of multiple motor function assessments and wearable sensors to measure disease progression, and the standardization of assessment conditions across visits (e.g., time of day, assistive device, hallway length), which enhances measurement consistency and reliability.

## Study limitations

Several important limitations affected this study. First, technical issues with the wearable sensors resulted in substantial data loss that directly impacts the feasibility assessment. Across all measurements, 13–47% of expected observations were missing due to device initialization failures, connectivity issues, and post-processing algorithm failures. This loss rate is particularly concerning for sit-to-stand transitions (22–33% missing) and suggests significant barriers to routine clinical implementation. Two participants (13% of enrolled) were excluded entirely due to device malfunctions, and among those included, missing data varied by parameter and time point.

Second, the small sample size (n = 10) limited statistical power to detect clinically meaningful changes and prevented subgroup analyses based on assistive device use or disease severity.

Third, the modified 6MWT corridor length (39 meters vs. standard 30 meters) may affect direct comparison with published reference equations and normative values, requiring cautious interpretation when comparing our 6MWD results to literature-based reference equations derived from the standard 30-meter course.

Fourth, the twelve-month observation period may have been insufficient to capture the full trajectory of disease progression in this slowly progressive condition, though our observed 4% decline in 6MWD aligns with published annual decline rates.

## Conclusions

This pilot study demonstrates that wearable sensors can capture gait and balance parameters during clinical TUG and 6MWT assessments in individuals with SBMA, with sensor-derived metrics showing strong correlations with standard functional measures. The sensors are easy to initialize during clinic visits, quick to place on the participant, not burdensome to the participant, and do not affect performance. However, substantial technical challenges remain before these tools are ready for routine clinical implementation. Data loss rates of 13–47% due to hardware failures, connectivity issues, and algorithm limitations represent a significant barrier that must be addressed through improved device reliability and disease-specific algorithm optimization.

Further longitudinal studies with larger sample sizes should examine prospective sensor measurements, include instrumented balance measures, evaluate subgroups based on assistive device use, and separately assess lower and upper limb function. Critically, future work must prioritize improving technical reliability and reducing missing data rates to make these sensors viable for clinical trials and remote monitoring in SBMA. Home monitoring could reduce participant burden and enable longer observation periods, but only if technical reliability substantially improves from the rates observed in this controlled clinical setting.

### Future directions

As digital tools become more common in neuromuscular research, new ways of analyzing data may help us spot very small movement changes in people with SBMA. In our study, we used basic statistics, but wearable sensors collect very detailed data that can be examined with more advanced computer models. One example is the Autoencoder–Gated Recurrent Unit (AE-GRU) model [38]. This type of model can simplify complex walking data and then look for patterns over time. In other fields, it has been very good at finding small changes that are hard to see. Using a model like this for SBMA could help detect tiny shifts in walking or balance before they are noticeable in regular clinic tests. In the future, applying these methods to data collected at home—where people walk more and in different ways—may help us catch early changes in function and develop better measures for testing‌‌ new treatments.

## Supporting information

S1 Data The de-identified data supporting the findings of this study have been provided as supporting information. (XLSX)
